# Promotion of epithelial-mesenchymal transformation by hepatocellular carcinoma-educated macrophages through Wnt2b/β-catenin/c-Myc signaling and reprogramming glycolysis

**DOI:** 10.1186/s13046-020-01808-3

**Published:** 2021-01-06

**Authors:** Yu Jiang, Qiuju Han, Huajun Zhao, Jian Zhang

**Affiliations:** Institute of Immunopharmaceutical Sciences, School of Pharmaceutical Sciences, Shandong University, 44 West Wenhua Road, Jinan, 250012 Shandong Province China

**Keywords:** Tumour-associated macrophages, Hepatocellular carcinoma, Epithelial-mesenchymal transformation, Tumour microenvironment, Glycolysis, Wnt2b, β-Catenin, C-Myc

## Abstract

**Background:**

Tumour-associated macrophages (TAMs) in the tumour microenvironment (TME) can promote the progression of hepatocellular carcinoma (HCC). Some tumours can be suppressed by targeting Wnt2b in tumour cells. However, the role of Wnt2b in HCC is still unknown. In particular, the role of Wnt2b-mediated signal activation in macrophage polarization in the HCC microenvironment, and the regulatory effect between Wnt and glycolysis in TAMs has not been described.

**Methods:**

The expression of Wnt2b in TAMs was detected by qPCR and immunofluorescence. Wnt2b/β-catenin interference in HCC-TAMs was performed by lentivirus carrying targeted shRNA or TLR9 agonist. Markers related to macrophage polarization and the changes of key glycolytic enzymes expression were detected by flow cytometry and qPCR. ECAR was analysed by Seahorse analyser. MTT assay, wound healing assay, western blotting were used to evaluate the promoting effect of different HCC-TAMs on the proliferation, migration and EMT of HCC in vitro. Tumour cells and different HCC-TAMs were injected via subcutaneously into immunodeficient mice to assess the effects of CpG ODN, Wnt2b, or β-catenin on HCC-TAMs in tumour growth in vivo.

**Results:**

Polarization-promoting factors derived from HCC cells upregulated the expression of Wnt2b in macrophages, which promoted the polarization of TAMs to M2-like macrophages by activating Wnt2b/β-catenin/c-Myc signalling. Furthermore, this process was associated with the activation of glycolysis in HCC-TAMs. These HCC-TAMs could promote the development of EMT, proliferation, and migration of HCC. In addition to silencing Wnt2b or β-catenin expression, TLR9 agonist CpG ODN downregulated the level of glycolysis and inhibited the M2 polarization of HCC-TAMs, reversing the tumour-promoting effects of TAMs in vitro and vivo.

**Conclusions:**

As a potential target for HCC therapy, Wnt2b may play an important regulatory role for the functions of TAMs in the TME. Moreover, the TLR9 agonist CpG ODN might act as a Wnt2b signal inhibitor and can potentially be employed for HCC therapy by disturbing Wnt2b/β-catenin/c-Myc and inhibiting glycolysis in HCC-TAMs.

## Background

The liver, an organ with immunological properties, is closely related to the proliferation and deterioration of tumour cells caused by oncogenic factors such as HBV and HCV infection, alcoholism, gene mutation, and abnormal activation of signaling pathways. Moreover, the occurrence and development of hepatocellular carcinoma (HCC) is associated with the immunosuppressive tumour microenvironment (TME) in the liver. In addition to tumour cells, the TME mainly consists of 1) cellular components such as tumour-associated fibroblasts, tumour-associated immune cells, and endothelial cells and 2) non-cellular components such as cytokines and growth factors.

Macrophages—a type of tumour-associated inflammatory cell—are a major component of most solid tumours, including HCC. They represent up to 50% of the tumour mass in tumour tissues of some patients [1]. These infiltrated macrophages are known as tumour-associated macrophages (TAMs), which are mainly differentiated from peripheral circulating monocytes rather than the expansion of resident macrophages in tumour tissue [2]. Importantly, these macrophages can be polarized to different phenotypes based on the microenvironment of different diseases; thus, displaying different roles in HCC.

Polarized macrophages are generally classified into two major groups: 1) classically activated macrophages (M1), exhibiting pro-inflammatory effects, and 2) alternatively activated macrophages (M2), exhibiting anti-inflammatory effects. Polarized macrophages can be identified by distinctive surface markers and functional molecules. TAMs with tumour-killing effects show phenotypic characteristics similar to those of M1-type macrophages, while TAMs with tumour-promoting effects show phenotypic characteristics similar to those of M2-type macrophages. Ample studies have indicated TAMs to be primarily M2 polarized, which can be induced by tumour-derived signals. TAMs and HCC cells regulate each other as TAMs promote the proliferation, metastasis, development of stemness, drug resistance, and angiogenesis of HCC, and inhibit adaptive immunity against HCC [3]. HCC cells can induce the M2 polarization of TAMs through the upregulation of B7-H3 or expression of Tim-3 in macrophages [4, 5]. However, the underlying mechanisms involved during the interaction between HCC cells and TAMs have not been fully clarified.

Both M1- and M2-like TAMs exhibit great plasticity in their phenotype and function. Various soluble factors (IL-10, TGF-β, IL-4, etc.) in the TME play an important role in the polarization and functional regulation of TAMs [6]. Toll-like receptors (TLRs)—part of the pattern recognition receptors—are mainly expressed in macrophages and other innate immune cells. TLR7/8 agonists are able to reverse the M2 polarization of colon carcinoma-associated macrophages and increase the sensitivity of tumour cells to chemotherapy drugs [7]. Moreover, Yang et al. [8] designed a safe and highly specific TLR2 agonist that is able to induce M1 polarization of TAMs to exert an anti-tumour effect; thus, demonstrating the role of TLR agonists in regulating the polarization of TAMs. Additionally, the transformation of different metabolic patterns may be related to the differentially activated phenotypes of macrophages [9]. For instance, in classical LPS/IFN-γ-activated M1 macrophages, glycolysis, pentose phosphate pathway, and fatty acid synthesis are enhanced. In IL-4/IL-13-activated M2 macrophages, oxidative phosphorylation, and fatty acid oxidation are enhanced [10].

As a secreted protein, Wnt ligands mediate a highly conserved signaling pathway during biological evolution; thus, playing an essential role in many physiological and pathological processes, especially in the development of tumours [11, 12]. Aberrant activation of the Wnt/β-catenin signalling pathway has been observed in HCC patients which is closely related to the procession of HCC [13, 14]. Specifically, the activation of Wnt signaling has been reported to enhance glycolysis in tumour cells and play an important role in the formation of a tumour immunosuppressive microenvironment by mediating cell-cell interactions [15]. Studies have shown Wnt3a to promote M2 polarization by activating β-catenin in macrophages [16]. Additionally, the expression of Wnt5a in TAMs promotes tumour invasion by activating non-classical Wnt signalling in breast cancer cells [17]. Although tumour suppression by targeting Wnt2b has been found in a variety of tumours [18], the role of Wnt2b—despite having an important role in the Wnt family—in HCC is still unknown. In particular, the role of Wnt2b-mediated signal activation in macrophage polarization in the HCC microenvironment, and the regulatory effect between Wnt and glycolysis in TAMs has not been described.

In this study, we described the polarized phenotype of HCC-educated macrophages (HCC-TAMs) and the role of these macrophages in promoting the development of epithelial-mesenchymal transformation (EMT) in HCC. Furthermore, during this process, we clarified the mechanism of the Wnt2b/β-catenin signalling pathway. Our findings reveal a novel mechanism in the complex regulatory network between HCC cells and macrophages as well as the formation of an immunosuppressive microenvironment in tumour tissues.

## Methods

### Cell lines and reagents

Human hepatoma cell lines Huh-7 (from the Chinese Academy of Sciences, Shanghai, China) and SMMC-7721 (from Shandong University Affiliated Shandong Cancer Hospital and Institute, Jinan, China) were conserved in our laboratory. The HCC cells were cultured in DMEM and RPMI 1640 medium (Hyclone, Logan, Utah, USA), respectively, while the human acute monocytic leukaemia cell line THP-1 (from the Chinese Academy of Sciences) was cultured in RPMI 1640 medium (Hyclone). All cultures were supplemented with 10% fetal bovine serum (FBS), 100 U/mL penicillin and 100 mg/mL streptomycin and maintained in a 5% CO_2_ incubator at 37 °C. The TLR9 agonist CpG OND (ODN M362, tlrl-m362–1) was purchased from InvivoGen (San Diego, California, USA). Phorbol-12-myristate-13-acetate (PMA) was purchased from Beyotime (S1819; Shanghai, China). 2-Deoxy-D-glucose (2DG, D8375) was purchased from Sigma-Aldrich (St Louis, MO, USA). The human cytokines M-CSF, IL-10, and TGF-β were purchased from PeproTech (Rocky Hill, NJ,USA).

### Inducing the PBMC-M, THP-1-M, HCC-TAMs and condition medium (CM)

Peripheral blood mononuclear cells (PBMCs) were derived from peripheral blood of healthy donors using the Human Blood Mononuclear Cell Separation Kit (P9010, Solarbio, Beijing, China). To obtain PBMC-derived macrophages (PBMC-M), CD14^+^ monocytes were derived from PBMCs using the CD14 Monocyte Isolation Kit (130–050-201; Miltenyi Biotec, Bergisch Gladbach, Germany) and induced with 100 ng/mL M-CSF for 5 days. Following treatment with 100 ng/mL PMA for 24 h, THP-1 cells were rested for another 24 h in RPMI 1640 medium to obtain THP-1-derived macrophages (THP-1-M). HCC-TAMs were generated following the incubation of these macrophages in 50% HCC-TCM for 48 h. The indicated macrophages were washed with 1× PBS for three times and incubated in RPMI 1640 for another 24 h to obtain the condition medium (CM).

### Patient samples

Peripheral blood samples from healthy donors were obtained from Qilu Hospital in accordance with the Ethics Committee of Shandong University, China, and informed consent was obtained from all participants. For the studies on human tissue samples, a tissue microarray (TMA) containing pairs of tumours and matched para-carcinoma tissues of 10 HCC patients were constructed by Shanghai Biochip Co., Ltd.(China), and approved by the Ethical Committee of Zhejiang province Taizhou Hospital. Written informed consent was obtained from all subjects in this study.

### HCC-conditioned medium (HCC-TCM)

HCC cells were grown to 70% confluence in the appropriate complete medium in 10-cm tissue culture dishes for 48 h. The supernatants were collected and centrifuged at 3000 g for 10 min for removal of dead cells and cell debris. The supernatant was then filtered through a 0.22 μm filter purchased from Millipore (Boston, MA, USA) and stored at − 80 °C until further use.

### Cell proliferation assay

3-(4,5-dimethyl-2-thiazolyl)-2,5-diphenyl-2-H-tetrazolium bromide (MTT, 5 mg/mL, M8180; Solarbio) assay was used to evaluate cell growth according to the manufacturer’s instructions. Briefly, HCC cells were plated in 96-well plates. With the attachment of the cells to the plate, the supernatant was removed, and the indicated CM was added. After 24 h, 20 μL MTT solution was added to each well and incubated at 37 °C. After 4 h, the supernatant was removed and 150 μL DMSO was added to each well to dissolve the formazan crystals. The absorbance at 490 nm was then determined using a scanning multiwall spectrophotometer (Bio-Rad, Hercules, CA, USA).

### Wound healing assay

The HCC cells were plated onto 24-well plates and incubated in RPMI 1640 or DMEM containing 10% FBS until sub-confluence was reached. Wound healings were made to the cell monolayer using a 200 μL plastic pipette tip. The cells were then cultured in the indicated medium. After 24 h, the wound healing areas were captured using a microscope (IX73, Olympus, Tokyo, Japan). The wound distance was measured and averaged from 3 points per wound area using the Image Pro Plus software. The experiments was performed in triplicate.

### RNA isolation and quantitative real-time PCR

Total RNA was extracted using Trizol reagent (Invitrogen, Carlsbad, California) and then used to synthesize cDNA using the FastQuant RT Kit (KR106; TIANGEN, Beijing, China). Sequences of the primers used for the PCR analysis are listed in Table S1. Quantitative PCR was performed according to a standard protocol using the SYBR Green Real-Time PCR MasterMix (Roche, Basel, Switzerland) in a Roche LightCycler 96 System. The PCR conditions were: 94 °C for 180 s, followed by 50 cycles of 94 °C for 10 s, 65 °C for 15 s, and 72 °C for 15 s; followed by 95 °C for 10 s, 65 °C for 60 s, and 97 °C for 1 s. Relative mRNA expression levels of the gene of interest were calculated using the 2^-△△Ct^ method.

### Plasmids and cell transfection

Active Wnt2B-V5 encoding homo Wnt2b (plasmid #43808; Addgene) was a gift from Xi He (Professor of Neurology, Harvard Medical School, USA). The control vectors were conserved in our laboratory. In the overexpression experiment, THP-1-M cells were transfected with 1 μg plasmid vector mixed with Lipofectamine 2000 reagent (11668–019, Invitrogen, CA, USA) according to the manufacturer’s instructions. The human beta-catenin (CTNNB1) shRNA lentivirus vectors pLKO.1-sh-β-catenin and its control vectors were purchased from Addgene (Plasmid #19761). The human Wnt2b shRNA lentivirus vectors pLKO.1-sh-Wnt2b were generated via ligation of lentivirus vector pLKO.1-puro with oligonucleotides, and the correct insertion was confirmed by sequencing. The targeting sequence for human Wnt2b gene interference was as follows: CGGCGGACTGATCTTGTCTACTTTCTCGAGAAAGTAGACAAGATCAGTCCGTTTTTG. Lentiviruses with pLKO.1-sh-Wnt2b or pLKO.1-sh-β-catenin were generated to knock down Wnt2b or β-catenin expression. Briefly, 293 T cells were co-transfected with pLKO.1-sh-β-catenin or pLKO.1-sh-Wnt2b and the packaging vectors pMD2G and pSAX2 using calcium phosphate transfection. After 48 h, the supernatants were collected, filtered through a 0.45 μm filter, and stored at − 80 °C until further use. THP-1 cells were transduced using the lentivirus supernatants with 10 μg/mL polybrene (Sigma-Aldrich) for 8 h. After washing twice, the cells were cultured with 1.5 μg/mL puromycin (Sigma-Aldrich) for another 5 days prior to use.

### Western blot analysis

Cells were lysed with RIPA peptide lysis buffer (P0013B; Beyotime, Shanghai, China) and centrifuged at 13,000 g for 10 min. The resulting supernatants were used for western blotting. Nuclear and cytoplasmic proteins were extracted using the Nuclear and Cytoplasmic Protein Extraction Kit (P0028; Beyotime, Shanghai, China) according to the manufacturer’s instructions. Protein concentrations were determined using an Enhanced BCA Protein Assay Kit (P0010S; Beyotime). Protein extracts were separated by 10% SDS-PAGE and probed with the following antibodies (1:1000): E-cadherin (cat. no. 3195; Cell Signaling Technology, Danvers, MA, USA), N-cadherin (cat. no. 13116; Cell Signaling Technology), vimentin (cat. no. 5741; Cell Signaling Technology), Snail (cat. no. 3879; Cell Signaling Technology), Twist (cat. no. A7314; ABclonal, Wuhan, China), ZEB1(cat. no. ab203829; Abcam), Slug (cat. no. 9585; Cell Signaling Technology), β-catenin (cat. no. 8480; Cell Signaling Technology), β-actin (cat. no. AC026; ABclonal), LaminA/C (cat. no. 4777; Cell Signaling Technology). Antibody binding was revealed using an HRP-labelled goat anti-rabbit IgG (H + L) (A0208; Beyotime). The signals were visualised using a GelDoc XR+ imaging system (Bio-Rad). Integrated density of E-cadhein and N-cadherin was analyzed by ImageJ software to quantified E-cadhein/ N-cadherin ratio.

### Immunofluorescence

Macrophages were fixed with 4% paraformaldehyde for 20 min at room temperature, washed three times with PBS, and blocked with Immunol Staining Blocking Buffer (P0102; Beyotime) for 1 h at 37 °C. They were then incubated with monoclonal antibodies against human β-catenin (cat. no. 8480; Cell Signaling Technology) at 4 °C overnight at a dilution ratio of 1:100, and washed thrice with PBS. The cells were then incubated with a DyLight 549 dye conjugated goat anti-rabbit IgG (1:200; A23320; Abbkine, CA, USA) for 1 h. DAPI (C1002; Beyotime) was used for nuclear staining. Images were captured using a microscope (IX73, Olympus). TMAs containing pairs of tumours and matched para-carcinoma tissues of 10 HCC patients were used to compare the expression of Wnt2b in macrophages. Briefly, TMA was deparaffinized with xylene, rehydrated with ethanol, and incubated with Immunol Staining Blocking Buffer (P0102; Beyotime) and 5% goat serum (C0265; Beyotime), followed by incubation with primary antibodies against CD68 (1:100; cat. no. A13286; ABclonal) and Wnt2b (1:100; sc-166,502; Santa Cruz, CA, USA) at 4 °C overnight. Then, the TMAs were incubated with two different fluorochrome-conjugated secondary antibodies, goat anti-rabbit DyLight 488 dye (1:200; A23220; Abbkine) and goat anti-mouse Cy3 dye (1:200; cat. no. AS008; ABclonal), at 37 °C for 1 h. DAPI was used for nuclear staining. Images were captured using a microscope (IX73, Olympus).

### Flow cytometry

Cells were incubated with mouse serum for 30 min, and surface stained with anti-CD163 antibody (GHI/61; BioLegend, CA, USA) at 4 °C for 1 h, then washed with 1× PBS. All stained cells were measured using FACS Calibur (BD Biosciences, Franklin, NJ, USA). The data were analysed using the FCS Express V3 software.

### Tumour formation assay

Male BALB/c nu/nu mice (6 weeks old) were purchased from Beijing HFK Bioscience (Beijing, China) and housed under specific pathogen-free conditions. All animal protocols and experiments were approved by the Institutional Animal Care and Use Committee of Shandong University, China. All animals received humane care and all operations and experiments were performed according to the Guide for the Care and Use of Laboratory Animals. To assay the effects of CpG ODN, Wnt2b, or β-catenin on HCC-TAMs in tumour growth, tumour cells (6 × 10^6^) with or without the indicated HCC-TAMs (1.5 × 10^6^) were mixed with Matrigel (at a ratio of 4:1; Corning, Union City, CA, USA) in a total volume of 100 μL and then subcutaneously injected into the right subaxillary of 6-week old immunodeficient mice. Each group contained six animals. Six days after inoculation, tumour sizes were measured every 2 days using a Vernier calliper by two independent observers, and tumour volumes were calculated as volume (mm^3^) = a × b^2^/2 (a and b represent the largest and smallest tumour diameters).

### Immunohistochemistry (IHC)

Mouse tumour tissues were fixed with 4% paraformaldehyde for 24 h and embedded in paraffin. Sections (5 μm-thick) were prepared and blocked with Immunol Staining Blocking Buffer (P0102; Beyotime) and 5% goat serum (C0265; Beyotime) followed by incubation with primary antibodies against VIM (cat. no. 5741; Cell Signaling Technology), E-cadherin (cat. no. A3044; ABclonal), β-catenin (cat. no. 8480; Cell Signaling Technology) and c-Myc (cat. no. ab32072; Abcam) at 4 °C overnight. These sections were then washed and incubated with the mouse/rabbit streptomycin-biotin assay system (SP-9000; ZSGB-BIO, Beijing, China). Expression of vimentin and E-cadherin were visualised by 3,3′-diaminobenzidine tetrahydrochloride (ZLI-9017; ZSGB-BIO) staining. At least four random fields were examined for each sample. Images were captured using a microscope (IX73, Olympus).

### Extracellular acidification rate (ECAR) analysis

For the ECAR analysis, indicated THP-1-M was plated onto the Seahorse XFe24 Cell Culture Microplates (Agilent, Palo Alto, CA, USA). When cells were adhered, the medium was changed to Seahorse XF Base Medium (with 1 mM GlutaMax, pH 7.4; 102,353–100; Agilent) and incubated in a non-CO_2_ incubator for 1 h at 37 °C prior to the start of the assay. The assay was performed with Seahorse XF Glycolysis Stress Test Kit (103020–100; Agilent) according to the manufacturer’ s instructions. Briefly, the test consisted of four consecutive stages: basal (without drugs), glycolysis induction (10 mM glucose), maximal glycolysis induction (1 μM oligomycin), and glycolysis inhibition (50 mM 2DG). The data were collected and analysed using the XFe24 wave software (Agilent).

### GEPIA2 database and overall survival curve

Overall survival curves based on gene expression levels of HCC patients were drawn using the GEPIA2 database (http://gepia2.cancer-pku.cn/) based on the The Cancer Genome Atlas (TCGA) database via Kaplan–Meier analysis [19].

### Statistical analysis

GraphPad Software Prism 5.0 (San Diego, CA, USA) was used for statistical analysis. All data are presented as mean ± standard error of the mean (mean ± SEM). Comparisons were made with a standard two-tailed unpaired or paired t-test or one-way ANOVA with post hoc Bonferroni correction. All experiments were replicated at least two or three times, with similar results. Statistically significant differences were set at *p < 0.05, **p < 0.01, ***p < 0.001.

## Results

### HCC-TCM induces M2 polarization of macrophages

**Fig. 1:**
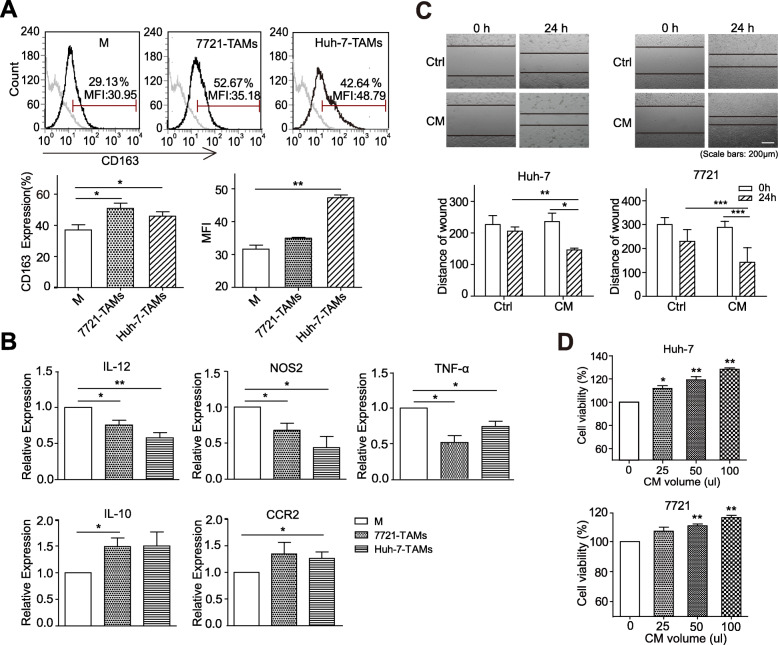
HCC-TCM induces M2 polarization of macrophages**.** THP-1 derived macrophages (THP-1-M) were incubated with 50% HCC-TCM for 48 h to obtain HCC-educated macrophages (HCC-TAMs). **a** The expression levels of CD163 on HCC-TAMs were detected by flow cytometry. **b** Transcript expression levels of markers for M1 or M2 macrophages were determined in HCC-TAMs by qPCR. HCC-TAMs were incubated with RPMI 1640 for an additional 24 h to obtain the condition medium (CM). **c** Huh-7 or SMMC-7721 cells were scratched with a plastic pipette tip and incubated with culture medium (Ctrl) or CM for 24 h. The results of this wound healing assay were photographed and measured. One representative of at least three independent experiments is shown. **d** Huh-7 or SMMC-7721 cells were incubated with the indicated volume of CM for 24 h. The cell viability of each group was detected by MTT assay. qPCR, quantitative real-time PCR; HCC, hepatocellular carcinoma; TCM, tumour condition culture medium; TAMs, tumour-associated macrophages; 7721, SMMC-7721. Data are presented as mean ± SEM from at least three independent experiments (*p < 0.05, **p < 0.01 and ***p < 0.001)

THP-1, a human mononuclear cell line, has been widely used to differentiate into macrophages; therefore, THP-1-M were selected for the relevant experiments in this study. First, THP-1-M were treated with HCC-TCM; flow cytometry analysis showed that TCM from different HCC cell lines could upregulate the expression of the M2-type macrophage marker CD163 to different degrees (Fig. 1a). Meanwhile, we also found that the mRNA levels of M1-type macrophage markers IL-12, NOS2, and TNF-α were downregulated, while M2-type macrophage markers IL-10 and CCR2 were upregulated in THP-1-M that was treated with HCC-TCM for 48 h (Fig. 1b). Similar results were observed in healthy human PBMC-derived macrophages (PBMC-M) (Supplementary Fig. 1). Recent studies have shown that TAMs show phenotypic characteristics similar to those of M2-type macrophages in tumours and play a significant role in tumour promotion [20]. To further verify if the TAMs induced by HCC-TCM (HCC-TAMs) have the characteristics of tumour-promoting TAMs, HCC cells were incubated with the CM of HCC-TAMs for 24 h. The CM of HCC-TAMs were found to significantly promote HCC migration (Fig. 1c) and promoted the viability of HCC cells in a concentration-dependent manner (Fig. 1d). These results suggested that the supernatant secreted by HCC could induce the polarization of macrophages to M2-like TAMs with tumour-promoting effects.

### HCC-TCM promotes M2 polarization via Wnt2b/β-catenin signalling

**Fig. 2:**
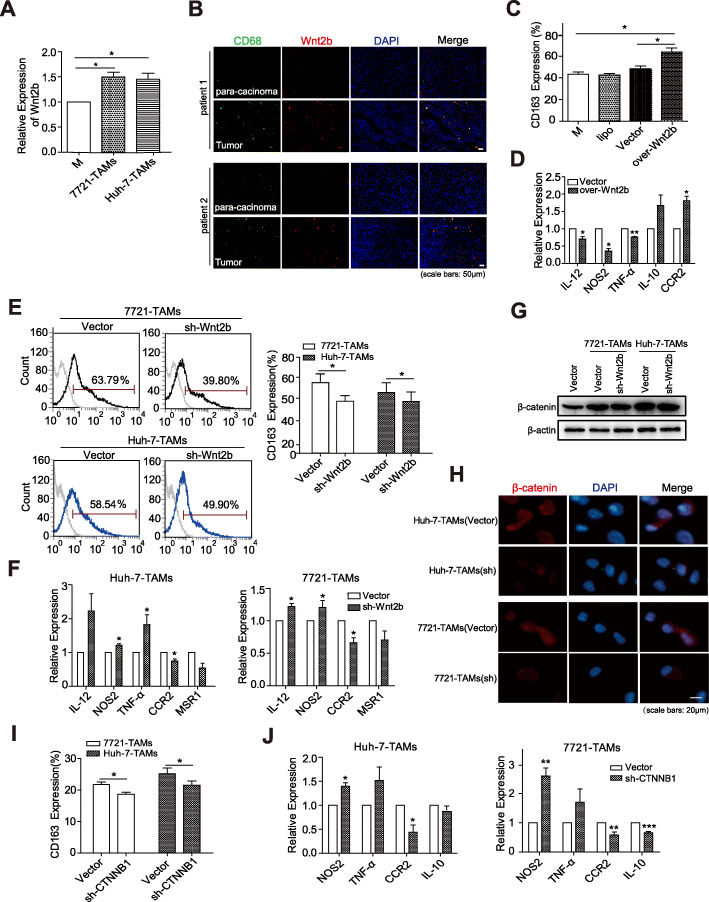
HCC-TCM promotes M2 polarization via Wnt2b/β-catenin signalling. **a** THP-1 derived macrophages (THP-1-M) were incubated with 50% HCC-TCM for 48 h to obtain the HCC-educated macrophages (HCC-TAMs). Transcript expression levels of Wnt2b were determined in HCC-TAMs by qPCR. **b** The expression levels of Wnt2b (red) in CD68^+^ macrophages (green) were determined by immunofluorescence using TMA containing pairs of tumors and matched para-carcinoma tissues of HCC patients. **c**, **d** THP-1-M were transfected with control vectors or Wnt2B-V5 (over-Wnt2b) vectors for 48 h. The expression levels of CD163 and markers for M1 or M2 macrophages on/in these cells were determined by flow cytometry and qPCR, respectively. THP-1-M infected with control vectors, sh-Wnt2b or sh-CTNNB1 (β-catenin) vector were acquired as described in the Materials and Methods, and then incubated with 50% HCC-TCM for 48 h. The expression levels of CD163 and markers for M1 or M2 macrophages on/in these cells were determined by flow cytometry (**e**, **i**) and qPCR (**f**, **j**), respectively. The expression levels of β-catenin in HCC-TAMs that were infected with control vectors or sh-Wnt2b vectors were determined by western blotting and immunofluorescence respectively (**g**, **h**). One representative of at least three independent experiments is shown. qPCR, quantitative real-time PCR; HCC, hepatocellular carcinoma; TCM, tumour condition culture medium; TAMs, tumour-associated macrophages; 7721, SMMC-7721; TMA, Tissue microarray. Data are presented as mean ± SEM from at least three independent experiments (*p < 0.05, **p < 0.01 and ***p < 0.001)

Recent studies have shown that high expression and activation of β-catenin in M2-type macrophages induced by IL-4, and Wnt ligands secreted by HCC could activate Wnt/β-catenin signalling in macrophages and induce M2-like TAMs development [16]. We found that TCM from different HCC cell lines could upregulate the expression of Wnt2b in macrophages to different degrees under the condition of M2 polarization of THP-1-M (Fig. 2a). In addition, fluorescence colocalization staining analysis for the TMA of HCC patients also showed that the expression of Wnt2b in macrophages within the tumour tissues was higher than that in adjacent tissues (Fig. 2b).

To clarify the role of Wnt2b in macrophage polarization, Wnt2b was overexpressed in THP-1-M. Compared to the control THP-1-M, CD163 expression was upregulated in THP-1-M Wnt2b-overexpressed cells (Fig. 2c), accompanied by the downregulation of IL-12, NOS2, and TNF-α, as well as the upregulation of IL-10 and CCR2 (Fig. 2d). On the contrary, as silencing Wnt2b by the lentivirus carrying targeted Wnt2b-shRNA, the expression of CD163 on THP-1-M induced by HCC-TCM was inhibited (Fig. 2e). Meanwhile, the expression of IL-12, NOS2, and TNF-α was upregulated, but the expression of MSR1 and CCR2 was downregulated (Fig. 2f). These data indicated the participation of Wnt2b in the M2 polarization process induced by HCC-TCM.

The classical Wnt signalling regulates the expression of downstream genes by activating β-catenin. To confirm if the activation of β-catenin leads to the contribution of Wnt2b in the M2 polarization of TAMs, we examined the effect of silencing Wnt2b on the levels of β-catenin at the protein level. As shown in Fig. 2g the total level of β-catenin was increased in HCC-TAMs, but decreased with the silencing of Wnt2b. β-catenin levels in nuclear and cytoplasmic fractions were further analyzed by immunofluorescence (Fig. 2h) and western blotting (Supplementary Fig. 2A&B). The results showed that nuclear translocation of β-catenin was decreased in HCC-TAMs Wnt2b-silenced cells. Similarly, HCC-TCM treatment upregulated the expression of Wnt2b, CTNNB1 (β-catenin) and Axin-2, an universal target molecule of Wnt/beta-catenin signal in PBMC-M (Supplementary Fig. 2C-E). Furthermore, silencing CTNNB1 with the lentivirus carrying targeted CTNNB1-shRNA inhibited HCC-TCM-induced expression of CD163 on HCC-TAMs (Fig. 2i). Moreover, the expression of IL-12 and TNF-α was upregulated while the expression of IL-10 and CCR2 was downregulated in the sh-CTNNB1 HCC-TAMs (Fig. 2j). These findings demonstrate the ability of HCC-TCM in promoting M2 polarization by activating classical Wnt2b/β-catenin signalling in macrophages.

### The activation of Wnt2b/β-catenin signalling enhances TAMs-induced tumour-promoting effects

**Fig. 3:**
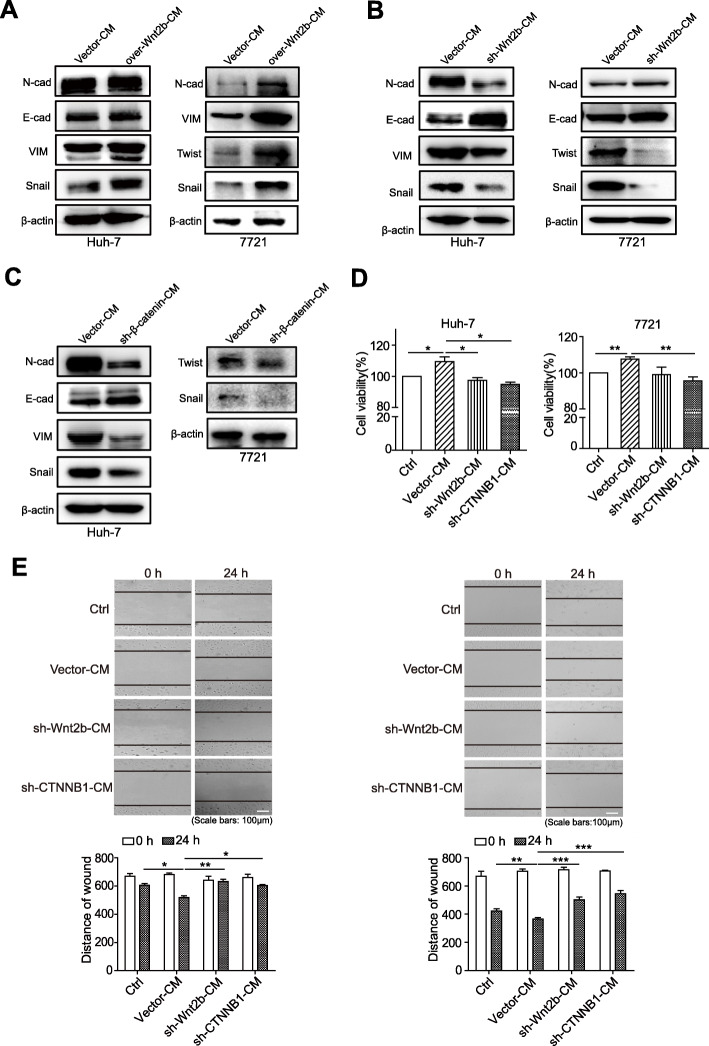
The activation of Wnt2b/β-catenin signalling enhances TAMs-induced tumour-promoting effects. **a** THP-1 derived macrophages (THP-1-M) were transfected with control vectors or Wnt2B-V5 (over-Wnt2b) vectors for 48 h. These macrophages were incubated with RPMI 1640 for an additional 24 h to obtain the condition medium (CM). HCC cells were cultured in the presence of indicated CM for 48 h. The expression levels of EMT markers were determined by western blotting. (**b-e**) THP-1-M infected with control vectors, sh-Wnt2b or sh-CTNNB1 (β-catenin) vectors were acquired as described in Materials and Methods. These macrophages were incubated with 50% HCC-TCM for 48 h for the preparation of the different TAMs. These TAMs were incubated with RPMI 1640 for another 24 h to obtain the CM. **b**, **c** HCC cells were cultured in the presence of the indicated CM for 48 h. The expression levels of EMT markers were determined by western blotting. **d** HCC cells were incubated with culture medium (Ctrl) or the indicated CM for 24 h. The cell viability of each group was detected by MTT assay. **e** HCC cells were scratched with a plastic pipette tip and incubated with culture medium (Ctrl) or indicated CM for 24 h. The results of this wound healing assay were photographed and measured. One representative of at least three independent experiments is shown. qPCR, quantitative real-time PCR; HCC, hepatocellular carcinoma; TCM, tumour condition culture medium; TAMs, tumour-associated macrophages; 7721, SMMC-7721. Data are presented as mean ± SEM from at least three independent experiments (*p < 0.05, **p < 0.01 and ***p < 0.001)

EMT, considered an important mechanism that promotes the progression of malignant tumours, is related to malignant biological behaviours such as proliferation and migration of tumours; TAMs facilitate tumour progression by promoting EMT [21]. We observed that the culture medium of Wnt2b-overexpressed macrophages could noticeably upregulate the expression of mesenchymal markers N-cadherin and vimentin, and the EMT-related transcription factors Snail, Twist and ZEB1 in HCC cells (Fig. 3a; Supplementary Fig. 3A), but downregulated the E-cadherin/N-cadherin ratio in HCC cells (Supplementary Fig. 3D). Consistently, compared with the HCC cells treated with the culture medium of control HCC-TAMs (Vector-CM), the expression of the mesenchymal markers and the EMT-related transcription factors was downregulated (Fig. 3b; Supplementary Fig. 3B) but E-cadherin/N-cadherin ratio was upregulated (Supplementary Fig. 3E) in HCC cells treated with the culture medium of Wnt2b-silenced HCC-TAMs (sh-Wnt2b-CM). These results suggest the influence of Wnt2b expression on the tumour-promoting effect of HCC-TAMs.

Next, the role of Wnt2b/β-catenin signalling in TAMs-induced HCC EMT was further verified by silencing CTNNB1 (β-catenin) expression. Compared with the HCC cells treated with the culture medium of control HCC-TAMs (Vector-CM), the expression of N-cadherin, vimentin, Snail, Twist and ZEB1 was decreased (Fig. 3c; Supplementary Fig. 3C) but E-cadherin/N-cadherin ratio was upregulated (Supplementary Fig. 3F) in HCC cells treated with the culture medium of CTNNB1-silenced HCC-TAMs (sh-CTNNB1-CM). In addition, compared with untreated HCC cells (Ctrl), Vector-CM promoted the proliferation and migration of HCC cells, which was obviously suppressed by the silencing of Wnt2b or CTNNB1 (Fig. 3d & e). These results suggest that Wnt2b/β-catenin signalling mediates HCC-TAMs-induced HCC EMT, and silencing the expression of Wnt2b or CTNNB1 can reverse the tumour-promoting role of TAMs.

### Wnt2b/β-catenin signaling augments the glycolysis of HCC-TAMs

**Fig. 4:**
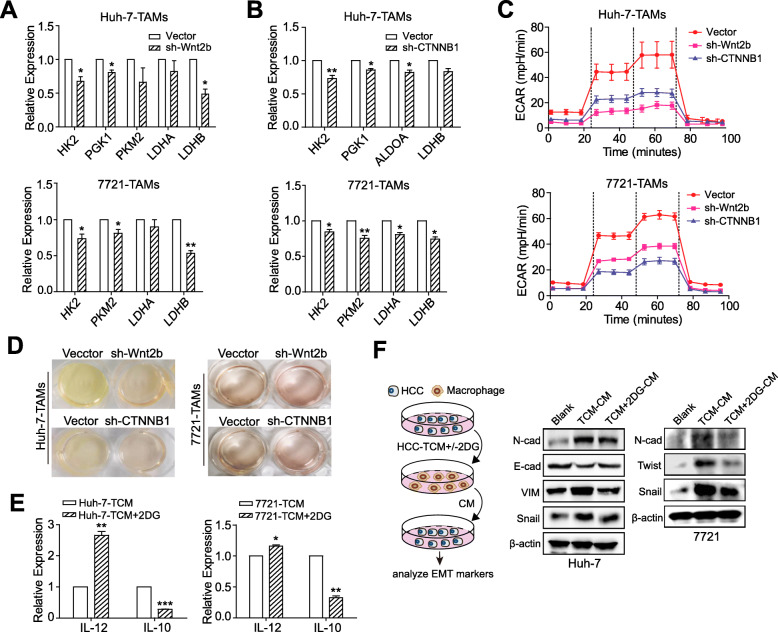
Wnt2b/β-catenin signals affect the glycolysis of HCC-TAMs. THP-1 derived macrophages (THP-1-M) infected with control vectors, sh-Wnt2b or sh-CTNNB1 (β-catenin) vectors were obtained as described in Materials and Methods. These macrophages were incubated with 50% HCC-TCM for 48 h to obtain the different TAMs. **a**-**b** The mRNA expression levels of key enzymes involved in glycolysis were determined in these cells by qPCR. **c** The extracellular acidification rate (ECAR) of the indicated TAMs was measured with a seahorse analyser. **d** Different TAMs were cultured under normoxic conditions for 24 h. Acidification of the culture medium was evaluated by visual inspection of the colour of the medium from the indicated TAMs. **e**-**f** THP-1-M were treated with HCC-TCM for 20 h in the presence or absence of 2DG (12.5 mM) to obtain different TAMs. The expression levels of polarization markers in these TAMs were determined by qPCR (**e**). These TAMs were incubated with RPMI 1640 for an additional 24 h to obtain the condition medium (CM). HCC cells were cultured in the presence of indicated CM for 48 h. The expression levels of EMT markers were determined by western blotting (**f**). 2DG, 2-deoxy-D-glucose; qPCR, quantitative real-time PCR; HCC, hepatocellular carcinoma; TCM, tumour condition culture medium; TAMs, tumour-associated macrophages; 7721, SMMC-7721. Data are presented as means ± SEM from at least two (**c**) or three (**a**, **b**, **d**-**f**) independent experiments (*p < 0.05, **p < 0.01 and ***p < 0.001)

An increasing number of studies have shown that glycolysis can also affect and regulate the phenotype and function of immune cells [9, 22]. To clarify if Wnt2b/β-catenin affected macrophage polarization by regulating glycolysis of HCC-TAMs, we first detected changes in the expression of key glycolytic enzymes in HCC-TAMs in which Wnt2b or CTNNB1 expression was silenced. The results showed that compared with the control HCC-TAMs, the expression of key glycolytic enzymes such as HK2, PGK1, PKM2, LDHA, and LDHB was downregulated in HCC-TAMs silenced Wnt2b or CTNNB1(Fig. 4a & b). In addition, the ECAR of HCC-TAMs was remarkably decreased by the silencing of Wnt2b or CTNNB1 compared with the control group (Fig. 4c). These observations were further confirmed by the visual inspection of the colour of the culture medium to evaluate acidification (Fig. 4d). Notably, we found that the glycolytic inhibitor 2DG upregulated the expression of IL-12 while downregulating the expression of IL-10 in HCC-TAMs (Fig. 4e), and blocked the ability of HCC-TAMs to induce HCC EMT (Fig. 4f; Supplementary Fig. 4A) accompanied with upregulated E-cadherin/N-cadherin ratio in HCC cells (Supplementary Fig. 4B). These findings indicate the involvement of Wnt2b/β-catenin signalling in glycolysis regulation and the tumour-promoting effects of HCC-TAMs.

### Wnt2b/β-catenin signalling in TCM-induced M2 polarization can be inhibited by the TLR9 agonist

**Fig. 5:**
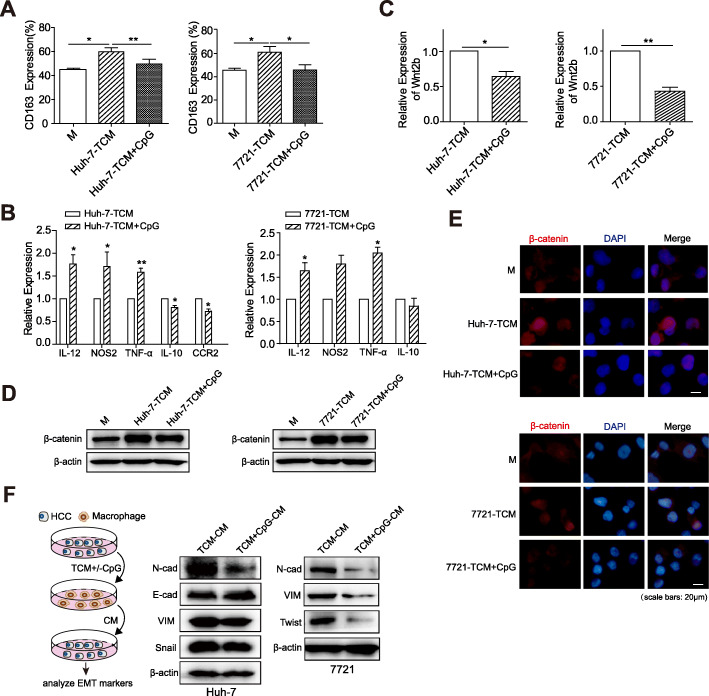
CpG ODN blocks TCM-induced M2 polarization and downregulates the expression of Wnt2b/β-catenin in HCC-TAMs. THP-1 derived macrophages (THP-1-M) were treated with HCC-TCM for 48 h in the presence or absence of CpG ODN (2 μg/mL). **a**, **b** The expression levels of CD163 and markers for M1 or M2 macrophages on/in these TAMs were determined by flow cytometry and qPCR, respectively. **c** The expression levels of Wnt2b in these TAMs were determined by qPCR. **d**, **e** The expression levels of β-catenin in THP-1-M and indicated TAMs were determined by western blotting and immunofluorescence, respectively. **f** These TAMs were incubated with RPMI 1640 for another 24 h to obtain the condition medium (CM). HCC cells were cultured in the presence of indicated CM for 48 h. The expression levels of EMT markers were determined by western blotting. One representative of at least three independent experiments is shown. qPCR, quantitative real-time PCR; HCC, hepatocellular carcinoma; TCM, tumour condition culture medium; TAMs, tumour-associated macrophages; 7721, SMMC-7721. Data are presented as means ± SEM from at least three independent experiments (*p < 0.05, **p < 0.01)

Studies on inflammation have shown that there is a certain interaction between TLRs and Wnt signalling [23]. Some experiments have validated that combined with the block in IL-10 receptor, the TLR9 agonist can reverse M2 polarization in breast cancer and colon cancer [24, 25]. Therefore, we analysed the effects of the TLR9 agonist CpG ODN on the polarization and Wnt signal activation of HCC-TAMs. The expression of CD163 on TAMs treated with TLR9 agonist CpG ODN was lower than that of TAMs treated with TCM alone (Fig. 5a). This was accompanied by the downregulation of IL-10 and CCR2, and the upregulation of IL-12, TNF-α, and NOS2 (Fig. 5b). Furthermore, we found that CpG ODN treatment decreased the expression of Wnt2b (Fig. 5c), the expression and nuclear translocation of β-catenin (Fig. 5d & e; Supplementary Fig. 5A&B), and the expression of Axin-2 in HCC-TAMs (Supplementary Fig. 5C). This phenomenon was also observed in human PBMC-M (Supplementary Fig. 6A-D). Notably, CpG ODN suppressed the ability of TAMs in promoting HCC EMT (Fig. 5f; Supplementary Fig. 5D) and upregulated E-cadherin/N-cadherin ratio in HCC cells (Supplementary Fig. 5E) These findings suggest that TLR9 agonists can inhibit the activation of Wnt2b/β-catenin signalling in HCC-TAMs, thereby inhibiting M2 polarization.

### The TLR9 agonist suppresses the glycolysis of HCC-TAMs via c-Myc

**Fig. 6:**
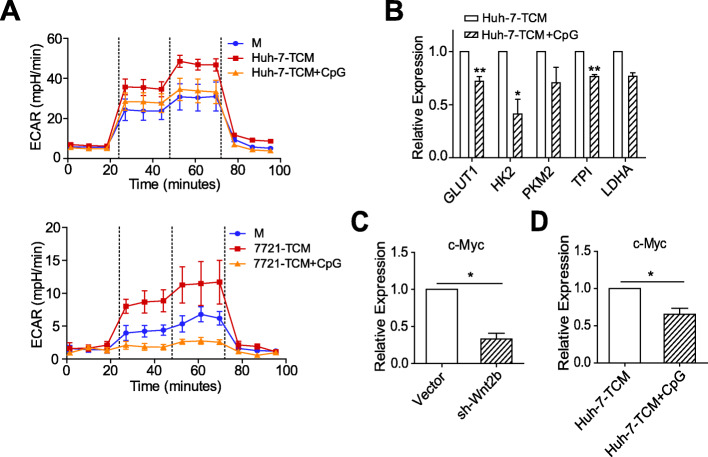
CpG ODN suppresses the glycolysis of HCC-TAMs via c-Myc. THP-1 derived macrophages (THP-1-M) were incubated with 50% HCC-TCM for 48 h in the presence or absence of CpG ODN (2 μg/mL). **a** The extracellular acidification rate (ECAR) of the indicated macrophages were measured with a seahorse analyser. **b** The mRNA expression levels of key enzymes involved in glycolysis were determined in Huh-7-TAMs by qPCR. **c**, **d** The mRNA expression levels of c-Myc were determined in Huh-7-TAMs by qPCR. qPCR, quantitative real-time PCR; HCC, hepatocellular carcinoma; TCM, tumour condition culture medium; 7721, SMMC-7721. Data are presented as mean ± SEM from at least two (**a**) or three independent experiments (**b**, **c**, **d**) (*p < 0.05, **p < 0.01)

As Wnt2b/β-catenin signalling affects the glycolysis level of HCC-TAMs, we further verified if CpG ODN could also inhibit the glycolysis of HCC-TAMs. As shown by Fig. 6a & b and Supplementary Fig. 6E, TCM upregulated the glycolysis level of macrophages, which was downregulated by CpG OND treatment. This was accompanied by the downregulation of key glycolytic enzymes. As an important downstream target gene of β-catenin, c-Myc is involved in Wnt2b/β-catenin signal transduction and acts as a key regulator of glycolysis in tumour cells [26]. We found that the expression of c-Myc in HCC-TAMs was inhibited by either the silencing of Wnt2b expression (Fig. 6c) or by CpG ODN treatment (Fig. 6d; Supplementary Fig. 6F). These results indicate that c-Myc mediates Wnt2b/β-catenin signal-regulated glycolysis of HCC-TAMs, which can be suppressed by TLR9 agonists.

### Inhibition of Wnt2b/β-catenin signaling reduces the tumour-promoting effect of HCC-TAMs in vivo

**Fig. 7:**
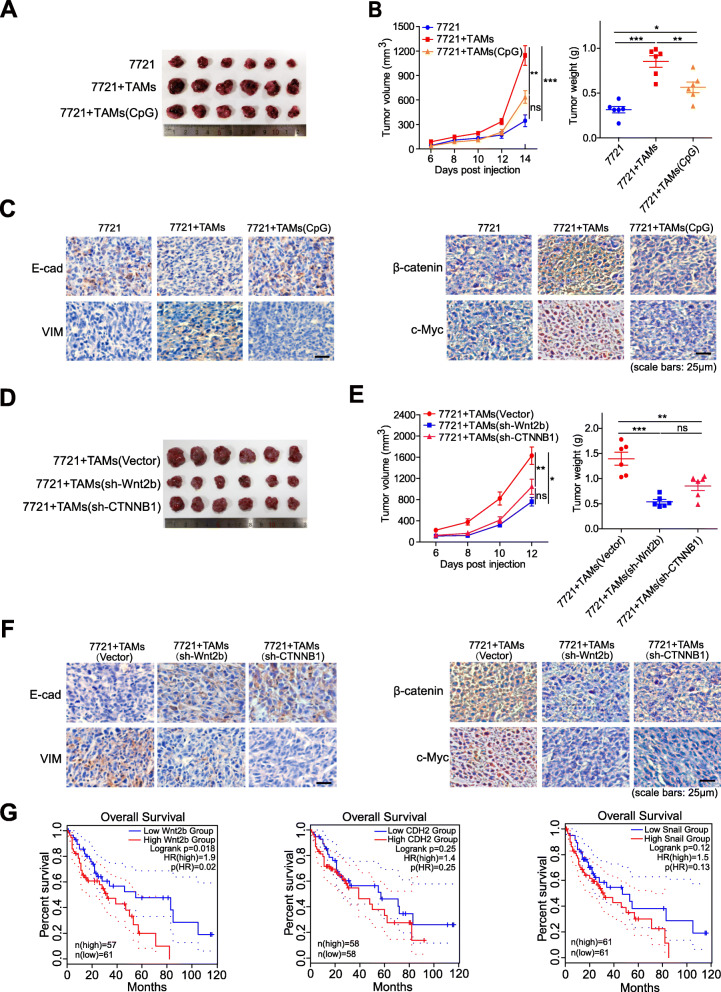
The inhibition of Wnt2b/β-catenin signaling reduces the tumour-promoting effect of HCC-TAMs in vivo. SMMC-7721 cells (6 × 10^6^) with or without the indicated TAMs (1.5 × 10^6^) were mixed with Matrigel (at ratio 4:1) and subcutaneously injected into the right subaxillary of 6-week old immunodeficient mice (*n* = 6 mice/group). **a**, **d** Representative images of the subcutaneous tumors from each group. **b**, **e** Growth of subcutaneous tumours (left); the average tumour weight of each group at the time of euthanisation(right). **c**, **f** Representative images of immunohistochemistry staining of vimentin, E-cadherin, β-catenin, c-Myc in tumour tissues. **g** Overall survival HCC patients related to indicated gene expression levels, were generated by the GEPIA2 database via Kaplan–Meier analysis. HCC, hepatocellular carcinoma; TAMs, tumour-associated macrophages; IHC, Immunohistochemistry. Data are presented as mean ± SEM (*p < 0.05, **p < 0.01 and ***p < 0.001)

SMMC-7721 cells alone or in combination with TAMs were inoculated subcutaneously into the right subaxillary of mice. We observed that the tumour growth rate was faster in mice inoculated with a mixture of SMMC-7721 cells and TAMs than in mice inoculated with SMMC-7721 cells alone, indicating that TAMs could promote HCC growth in vivo. However, TAMs-induced tumour-promoting effects could be suppressed by CpG ODN pre-treatment (Fig. 7a-b). Meanwhile, compared with tumour tissues from the inoculation of SMMC-7721 cells alone, the expression of E-cadherin was significantly downregulated, but the expression of vimentin, β-catenin and c-Myc was upregulated in tumour tissues that were inoculated from the mixture of SMMC-7721 and TAMs, which was reversed by CpG ODN pre-treatment of TAMs (Fig. 7c). Subsequently, compared with mice inoculated with a mixture of SMMC-7721 cells and TAMs that were transfected with a vector, we found that tumour growth rate and tumour load were remarkably reduced in mice inoculated with a mixture of SMMC-7721 cells and TAMs that had either silenced Wnt2b or CTNNB1 (Fig. 7d-e). Meanwhile, compared with tumour tissues from SMMC-7721 combined with TAMs transfected with a vector, the expression of E-cadherin was remarkably upregulated, but the expression of vimentin, β-catenin and c-Myc was downregulated in tumour tissues from SMMC-7721 mixed with silenced Wnt2b or CTNNB1 in TAMs (Fig. 7f). Finally, the GEPIA2 database was used to analyse the influence of Wnt2b expression on the prognosis of HCC patients. The overall survival of HCC patients with high expression of Wnt2b was significantly lower than that of the low expression group. In addition, the expression of EMT-related markers N-cadherin and Snail is also associated with poor prognosis of patients (Fig. 7g). These results demonstrate that Wnt2b/β-catenin signalling mediates TAMs-induced HCC-promoting effects, and TLR9 agonists might be a potential therapeutic agent.

## Discussion

With an integral role in the TME, TAMs can play a tumour-promoting role via interaction with HCC cells. Accumulation of M2-like TAMs is closely associated with poor prognosis in HCC patients [27]. Therefore, reversing the M2 polarization of TAMs may be a potential therapeutic target for the treatment of HCC. Although the mechanisms involved in inducing M2 polarization of TAMs have been widely reported, the mechanisms in regulating TAMs polarization have been continuously explored due to the complex regulatory network in the TME.

M2-type macrophages induced by IL-4/IL-13 are often utilized as TAMs to conduct experimental studies. Notably, compared with alternatively activated M2-type macrophages, TAMs formed in TME are regulated by more complex factors and have obvious heterogeneity [28]. Herein, to better mimic the characteristics of macrophages in the TME, we used the culture medium of HCC to “educate” macrophages, by showing similar phenotypic characteristics of M2-like TAMs. TAMs have been shown to promote tumour proliferation and migration, a process associated with EMT in tumour cells [29]. Similarly, the HCC-TAMs we used significantly promoted the EMT of HCC cells as well as the proliferation and migration of HCC cells, further proving that these HCC-TAMs display similar functional characteristics to M2-like TAMs.

Some studies have found that Wnt signalling plays an important role in tumour-induced immunosuppressive microenvironment formation [30]. Nevertheless because of the large number of ligand molecules in the Wnt family, the mechanisms of Wnt signalling activation in the TME also remain to be further explored—especially the role of Wnt signalling in HCC-TAMs polarization, which has completely not been reported. In a variety of Wnt ligands, compared with the other ligands, the role of Wnt3a in the regulation of TAMs polarization and function has been extensively studied [16, 31]. In this study, we first found that the expression of Wnt2b was upregulated in HCC-TAMs, and these tumour-promoting M2-like macrophages promote the proliferation, migration, and EMT of HCC. Further investigation proved that the upregulated Wnt2b can promote the polarization of HCC-TAMs by activating the classical Wnt/β-catenin pathway. The activation of Wnt/β-catenin signaling has been widely reported to be related to the occurrence and development of HCC. Notably, Tang et al. [32] have recently found that CTNND1(delta-catenin) was highly expressed in HCC tissues and dramatically enhanced Wnt/β-catenin signaling, indicating CTNND1 might act as a regulator of the canonical Wnt/β-catenin signaling pathway [33]. Although CTNND1 has been shown to be associated with macrophage migration and influence macrophage in the inflammatory response [34, 35], whether CTNND1 can promote the M2 polarization through activating canonical Wnt/β-catenin signaling pathway in HCC-TAMs remains to be further explored.

Various soluble factors in the TME can repolarize TAMs to M2-like macrophages through different mechanisms. Abnormal expression of IL-10 and TGF-β has been found in the HCC microenvironment [4, 36]. We observed that both IL-10 and TGF-β can stimulate the expression of Wnt2b, β-catenin, and c-Myc in macrophages (Supplementary Fig. 7), suggesting that various factors in TME mediate the polarization of HCC-TAMs via Wnt2b/β-catenin signalling.

Despite an adequate supply of oxygen, tumour cells select glycolysis as the main energy supply method—a phenomenon known as the Warburg effect—which promotes the occurrence and development of tumours [37]. Initially, the concept of Warburg effect was thought to be limited only to tumour cells, neglecting the metabolic interactions between tumour cells and other components. More recently, the emerging concept of the ‘reverse Warburg effect’ was proposed that stromal cells exhibit more aerobic glycolysis and metabolically supports adjacent tumour cells. The differential expression patterns of monocarboxylate transporters (MCT) in tumour cells and CAFs contribute to metabolic symbiosis, in which caveolin1-null CAFs tend to activate aerobic glycolysis and secrete lactate via MCT4; meanwhile, epithelial tumour cells express MCT1 that contributes to uptake of lactate, generating large amounts of ATP to meet the demands of development in tumour [38–40]. This effect evolved into dynamic interplay between tumour cells and tumour stromal compartments. In a complex TME, macrophages have unique metabolic characteristics and are involved in the metabolic reprogramming of the microenvironment. The polarization and function of macrophages are closely related to metabolism, and glucose metabolism is closely related to host defence among various metabolic modes [41]. In the TME, although TAMs have similar phenotypic characteristics to M2-type macrophages, there are differences in their selection of metabolic patterns. It has been demonstrated that TAMs are more inclined to select glycolysis as the main metabolic pattern [42] and inhibition of TAMs glycolysis can reverse the M2 phenotype [43, 44]. However, the regulatory roles of Wnt signalling and glycolysis in TAMs have not been described. In this study, we found that by silencing Wnt2b in macrophages the aerobic glycolysis level of HCC-TAMs can downregulated by blocking the activation of Wnt/β-catenin signalling and affecting M2 polarization. Similarly, the glycolytic inhibitor 2DG could antagonise the M2 polarization of macrophages induced by HCC-TCM, thereby blocking the intensive effect of HCC-TAMs on HCC EMT. Since the increased intratumoural infiltration of MCT4-positive macrophages has been suggested shorter overall survival in HCC patients [45], whether the ‘reverse Warburg effect’ also exists in HCC microenvironment between HCC cells and TAMs needs to be further explored.

As an oncogene, c-Myc is closely related to the development of human and mouse tumours. Moreover, activation of Wnt/β-catenin signalling can induce the expression of c-Myc [46]. A previous study has demonstrated that the upregulation of c-Myc expression is correlated with M2 polarization induced by IL-4, IL-10, TGF-β, and other factors [47]. We also found that the expression of c-Myc in macrophages could be upregulated by HCC-TCM, which suggested the involvement of c-Myc in the regulation of glycolysis in HCC-TAMs. It’s worth noting that endogenous expression of oncogenic c-Myc and Kras can increase the transcription of NRF2 to promote ROS detoxification, tumorigenesis [48] and tumour resistance [49]. NRF2 also plays a critical role in cancer metabolism by altering glucose and glutamine metabolis [50]. Tumour cell-derived lactate activates NRF2 in macrophages, skewing macrophage M2 polarization [51]. As the downstream regulator of c-Myc, NRF2 may also be involved in regulation of metabolism and polarization of HCC-TAMs, promoting tumorigenesis and tumour resistance by TAMs.

Metabolic checkpoint refers to a set of enzymes, receptors, transcription factors, and signaling complexes, which play important roles in a certain metabolic pathway and affect the immune function of immune cells [52]. As a typical metabolic checkpoint, mTOR plays a vital role in T cell differentiation and function [52]. mTOR inhibition mediates metabolic cell cycle checkpoints block, which can induce G1 cell cycle arrest [53, 54]. Based on the differences in the metabolic plasticity of effector T cells and tumour cells, blocking the glutamine metabolism checkpoint could cause a strong anti-tumor response [55]. In our study, we found that the HCC microenvironment made TAMs more prone to glycolytic metabolism and this process depended on the activation of Wnt2b/β-catenin/c-Myc. Inhibition of Wnt2b or β-catenin suppressed the glycolysis of TAMs and reversed its tumor-promoting effects. This suggests that in addition to key enzyme molecules during glycolysis, Wnt2b/β-catenin/c-Myc signalling might also act as an important metabolic checkpoint in HCC-TAMs. As a key enzyme and also another important metabolic checkpoint in glycolysis, the expression of PKM2 with lower glycolytic enzyme activity is necessary for the Warburg effect. On the one hand, it activates glycolysis to produce ATP more rapidly than the oxidative phosphorylation. On the other hand, the low glycolytic enzyme activity of PKM2 inhibits the conversion of pyruvate to lactate and promotes the production of glycolytic intermediates to enter the glycolysis branch pathways, thereby producing NADPH to protect tumour cells from accumulated ROS and involves in nucleotide synthesis [56]. Furthermore, c-Myc can promote glycolysis with up-regulation many glycolytic genes, including PKM2 [57–59]. The translocation of PKM2 into the nucleus could promote β-catenin transactivation, leading to the expression of c-Myc [60]. Therefore, a positive feedback loop between c-Myc and PKM2 might exist to drive glycolysis. In our study, we also observed the increased expression of c-Myc, PKM2 and other glycolytic genes in HCC-TAMs, indicating a similar regulatory effect may also exist between c-Myc and PKM2. However, the specific role of PKM2 in the glycolytic in HCC-TAMs remains to be further discovered.

Many studies have shown that a variety of TLR agonists can induce tumour suppression by triggering an anti-tumour immune response [61]. Among them, the TLR9 agonist showed improved therapeutic effects, whether used alone or in combination with other drugs, immunocheckpoint blocking therapy, or as an adjuvant of a tumour vaccine [62, 63]. In our previous work, we reported that the TLR9 agonist CpG ODN could promote the apoptosis of HCC [64]. But the role of TLRs in the polarization of HCC-TAMs is not completely clear. Interestingly, we found that the TLR9 agonist CpG ODN downregulated the expression of Wnt2b in HCC-TAMs and inhibited the activation of β-catenin, thus reversing the M2 polarization. Furthermore, CpG ODN could suppress the glycolysis of TAMs induced by HCC-TCM. Further investigation showed that silencing the expression of Wnt2b or using TLR9 agonist CpG ODN could both inhibit the activation of Wnt/β-catenin signalling and downregulate the expression of c-Myc in HCC-TAMs, inhibiting the glycolysis of TAMs and the process of M2 polarization induced by HCC-TCM. However, the specific mechanism by which CpG ODN regulates Wnt signalling remains to be further explored.

**Fig. 8:**
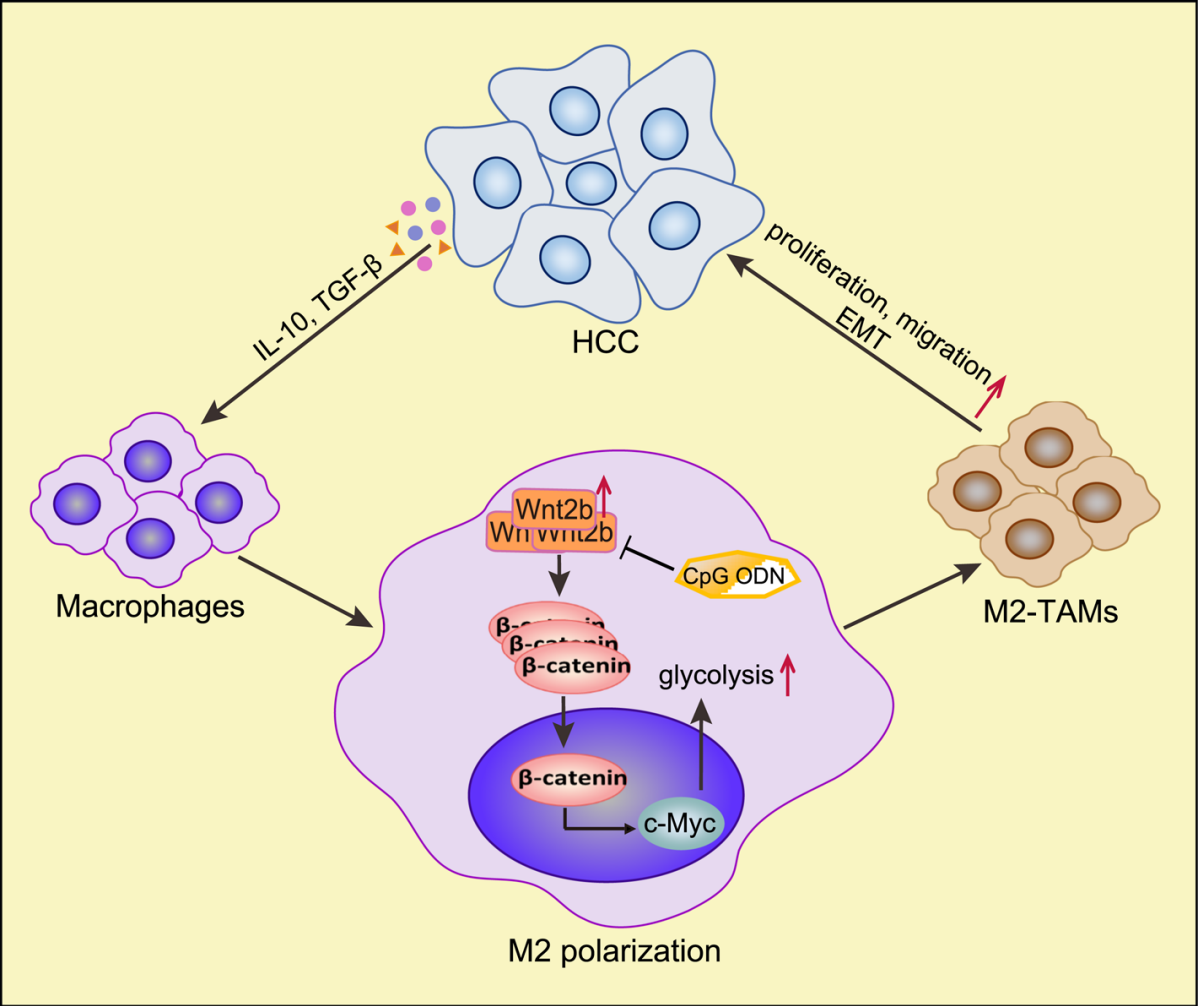
Schematic representation illustrates the positive feedback loop between HCC cells and HCC-TAMs. Polarization-promoting factors (IL-10, TGF-β, ect.) in the HCC TME can up-regulate the expression of Wnt2b in macrophages, then promote expression and nuclear translocation of β-catenin, which can promote the M2 polarization of TAMs, a process associated with the activation of HCC-TAMs glycolysis by activating c-Myc. These polarized TAMs can promote the proliferation, migration and EMT of tumor cells. TLR9 agonist CpG ODG can act as a blocker of Wnt2b signal which can inhibit M2 polarization of HCC-TAMs induced by HCC-TCM. HCC, hepatocellular carcinoma; TME, tumor microenvironment; TAMs, tumour-associated macrophages; EMT, epithelial-mesenchymal transformation

As shown in the schematic representation in Fig. 8, our current study illustrated that polarization-promoting factors in the HCC TME can activate β-catenin by upregulating the expression of Wnt2b in macrophages, which can promote the polarization of TAMs to M2-like macrophages—a process associated with the activation of HCC-TAMs glycolysis. The TLR9 agonist CpG ODG can act as an inhibitor of the Wnt2b signal, which can block the M2 polarization of HCC-TAMs induced by HCC-TCM. As a potential target of tumour therapy, Wnt2b can not only promote malignant formation of tumour cells, but also play an important role in the formation of an immunosuppressive tumour microenvironment.

## Conclusion

In summary, in this study, we investigated the role of Wnt2b-mediated signal activation in macrophage polarization in the HCC microenvironment, and the regulatory effect between Wnt and glycolysis in TAMs. Targeting Wnt2b in TAMs might be a great potential treatment strategy in immune therapy of HCC, and TLR9 agonist CpG ODN might act as a Wnt2b signal inhibitor for HCC therapy.

## Supplementary Information

**Additional file 1.**

## Data Availability

The dataset supporting the conclusions of this article is included within the article and its additional file.

## Abbreviations

TAMs: Tumour-associated macrophages
TME: Tumour microenvironment
HCC: Hepatocellular carcinoma
HCC-TAMs: HCC-educated macrophages
IFN-γ: Interferon-γ
IL-13: Interleukin-13
IL-4: Interleukin-4
IL-10: Interleukin-10
TGF-β: Transforming growth factor beta
TLRs: Toll-like receptors
EMT: Epithelial-mesenchymal transformation
FBS: Fetal bovine serum
PMA: Phorbol-12-myristate-13 acetate
2DG: 2-Deoxy-D-glucose
M-CSF: Macrophage colony stimulating factor
TMA: tissue microarray
TCM: tumour conditioned medium
THP-1-M: THP-1-derived macrophages
PBMCs: Peripheral blood mononuclear cells
PBMC-M: PBMC-derived macrophages
CM: Condition medium
NOS2: Nitric oxide synthase 2, inducible
TNF-α: Tumour necrosis factor alpha
HK2: Hexokinase 2
PGK1: Phosphoglycerate kinase 1
PKM2: Pyruvate kinase 2
ALDOA: Aldolase A
LDHA: Lactate dehydrogenase-A
LDHB: Lactate dehydrogenase-B
GLUT1: Glucose transporter type 1
TPI: Triosephosphate isomerase
