# Supplementary material for: Promotion of epithelial-mesenchymal transformation by hepatocellular carcinoma-educated macrophages through Wnt2b/β-catenin/c-Myc signaling and reprogramming glycolysis
Source: J Exp Clin Cancer Res. 2021 Jan 6;40:13. doi: 10.1186/s13046-020-01808-3 (PMC7788901; doi:10.1186/s13046-020-01808-3)
Supplement: Supplementary file 1 — Additional file 1. [file 13046_2020_1808_MOESM1_ESM.docx]

**Supplementary data to:**

**Promotion of epithelial-mesenchymal transformation by hepatocellular**

**carcinoma-educated macrophages through Wnt2b/β-catenin/c-Myc**

**signaling and reprogramming glycolysis**

Yu Jiang, Qiuju Han, Huajun Zhao, Jian Zhang

**Supplementary table S1**

Primer pairs used for quantitative real-time PCR analyze.

| **Gene** | **Sense primer (5'-3')** | **Antisense primer (5'-3')** |
| --- | --- | --- |
| IL-12  NOS2  TNF-α  IL-10  CCR2  MSR1  GLUT1  HK2  PGK1  PKM2  TPI  ALDOA  LDHA  LDHB  Wnt2b  CTNNB1  c-Myc  Axin-2  β-actin | TGCCTTCACCACTCCCAAAACC  GCTCTACACCTCCAATGTGACC  CTCTTCTGCCTGCTGCACTTTG  TCCCTGTGAAAACAAGAGCA  CAGGTGACAGAGACTCTTGGGA  TGCACAAGGCAGCTCACTTTGG  TTGCAGGCTTCTCCAACTGGAC  AAGCCCTTTCTCCATCTCCT  CTTGGGACAGCAGCCTTAAT  ATGGCTGACACATTCCTGGAGC GGCGAAGTCGATATAGGCAG  GACACTCTACCAGAAGGCGGAT  CGCCGATTCCGGATCTCATT  GCGTGTGCTATCAGCATTCTG  TGGATGCCAAGGAGAAGAGGCT  CACAAGCAGAGTGCTGAAGGTG  CCTGGTGCTCCATGAGGAGAC  ATTCGGCCACTGTTCAGACG  CACCATTGGCAATGAGCGGTTC | CAATCTCTTCAGAAGTGCAAGGG  CTGCCGAGATTTGAGCCTCATG  ATGGGCTACAGGCTTGTCACTC  ATAGAGTCGCCACCCTGATG  GGCAATCCTACAGCCAAGAGCT  GTGCAAGTGACTCCAGCATCTTC  CAGAACCAGGAGCACAGTGAAG  CTTCTTCACGGAGCTCAACC  CAAGCTGGACGTTAAAGGGA  CCTTCAACGTCTCCACTGATCG AGTTCTTCGTTGGGGGAAAC  GGTGGTAGTCTCGCCATTTGTC  AGCTGATCCTTTAGAGTTGCC A  TTCTCTGCACCAGATTGAGCC  GTACAGGAACCACTCACGCCAT  GATTCCTGAGAGTCCAAAGACAG  CAGACTCTGACCTTTTGCCAGG  GACAACCAACTCACTGGCCTG  AGGTCTTTGCGGATGTCCACGT |





**Supplementary Figure 1. HCC-TCM induces M2 polarization in human PBMC-M.** CD14^+^ cells were purified from the peripheral blood of healthy donors and induced with 100 ng/mL M-CSF for 5 days to obtain PBMC-derived macrophages (PBMC-M). PBMC-M were incubated with 50% Huh-7-TCM for 48 h. (A, B) The expression levels of CD163 and IL-12, IL-10 production on/in these cells were determined by flow cytometry and qPCR, respectively. qPCR, quantitative real-time PCR; PBMC, peripheral blood mononuclear cell; HCC, hepatocellular carcinoma; TCM, tumour condition culture medium. Data are presented as means ± SEM from at least three independent experiments (*p < 0.05).


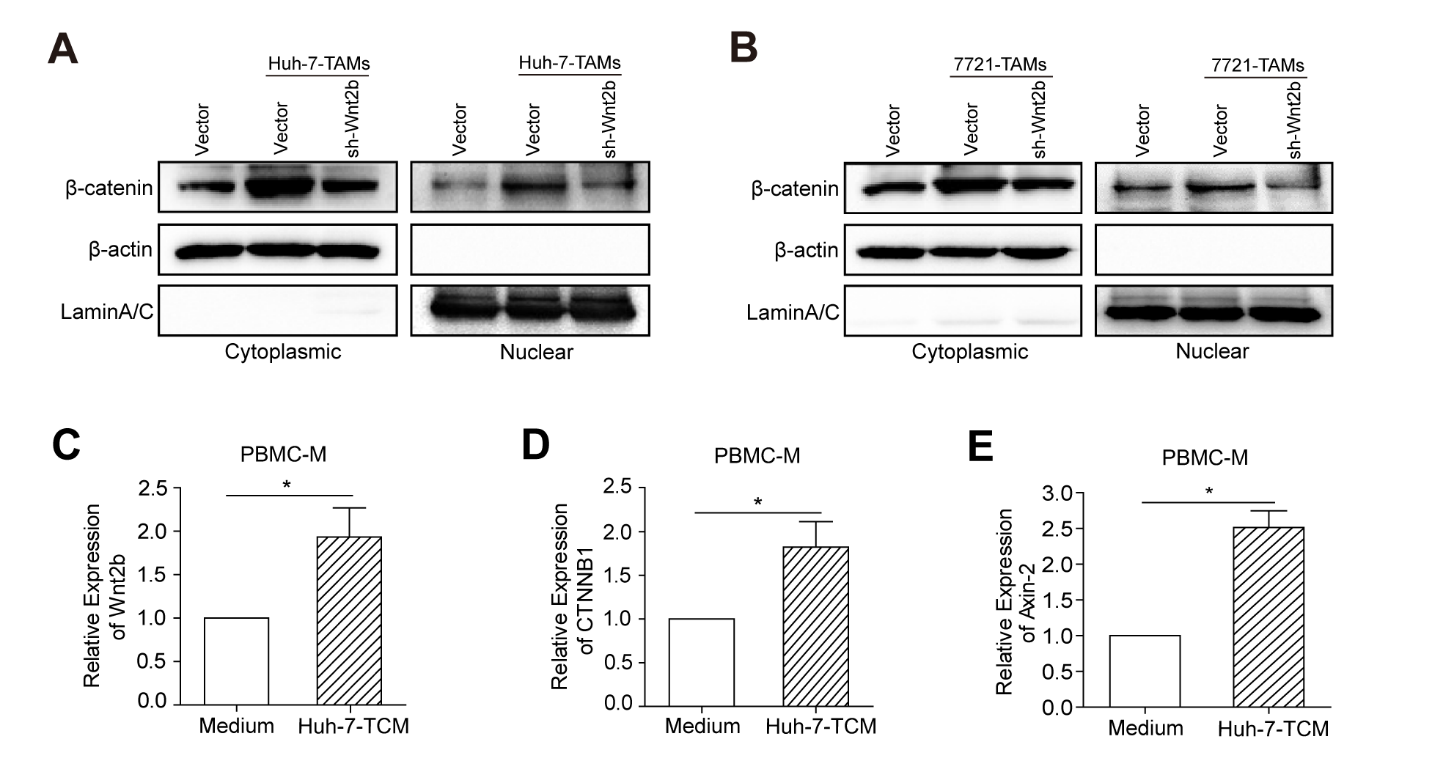


**Supplementary Figure 2. HCC-TCM activates Wnt2b/β-catenin signalling in THP-1-M and human PBMC-M.** (A, B) THP-1-M infected with control vector or sh-Wnt2b vector were acquired as described in the Materials and Methods, and then incubated with 50% HCC-TCM for 48 h. The cytosolic and nuclear proteins in these HCC-TAMs were extracted, and β-atenin levels were determined by western blotting. CD14^+^ cells were purified from the peripheral blood of healthy donors and treated with 100 ng/mL M-CSF for 5 days to obtain the PBMC-derived macrophages (PBMC-M). PBMC-M were incubated with 50% Huh-7-TCM for 48 h. The expression levels of Wnt2b (C), CTNNB1 (D) and Axin-2 (E) in these cells were determined by qPCR. One representative of at least three independent experiments is shown. qPCR, quantitative real-time PCR; PBMC, peripheral blood mononuclear cell; HCC, hepatocellular carcinoma; TCM, tumour condition culture medium; TAMs, tumour-associated macrophages; 7721, SMMC-7721. Data are presented as mean ± SEM from at least three independent experiments (*p < 0.05).


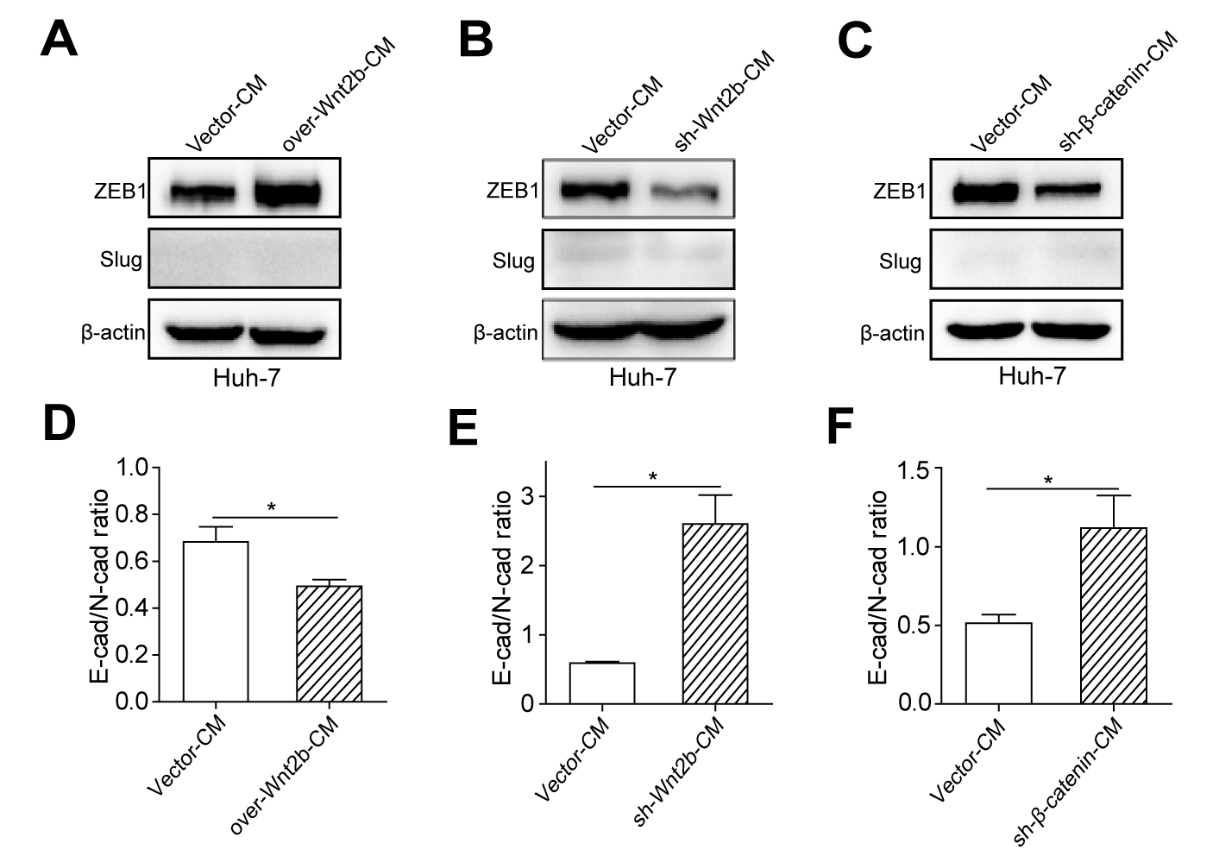


**Supplementary Figure 3. The activation of Wnt2b/β-catenin signalling in TAMs promotes EMT of HCC.** (A) THP-1 derived macrophages (THP-1-M) were transfected with control vectors or Wnt2B-V5 (over-Wnt2b) vectors for 48 h. These macrophages were incubated with RPMI 1640 for an additional 24 h to obtain the condition medium (CM). HCC cells were cultured in the presence of indicated CM for 48 h. The expression levels of ZEB1 and Slug were determined by western blotting. (B, C) THP-1-M infected with control vectors, sh-Wnt2b or sh-CTNNB1 (β-catenin) vectors were acquired as described in Materials and Methods. These macrophages were incubated with 50% HCC-TCM for 48 h for the preparation of the different TAMs. These TAMs were incubated with RPMI 1640 for another 24 h to obtain the CM. HCC cells were cultured in the presence of the indicated CM for 48 h. The expression levels of ZEB1 and Slug were determined by western blotting. (D-F) Statistical analysis of the E-cadhein/N-cadherin ratio in Huh-7 cells from Figure 3A, B, C was shown. One representative of at least three independent experiments is shown. TAMs, tumour-associated macrophages. Data are presented as mean ± SEM from at least three independent experiments (*p < 0.05).


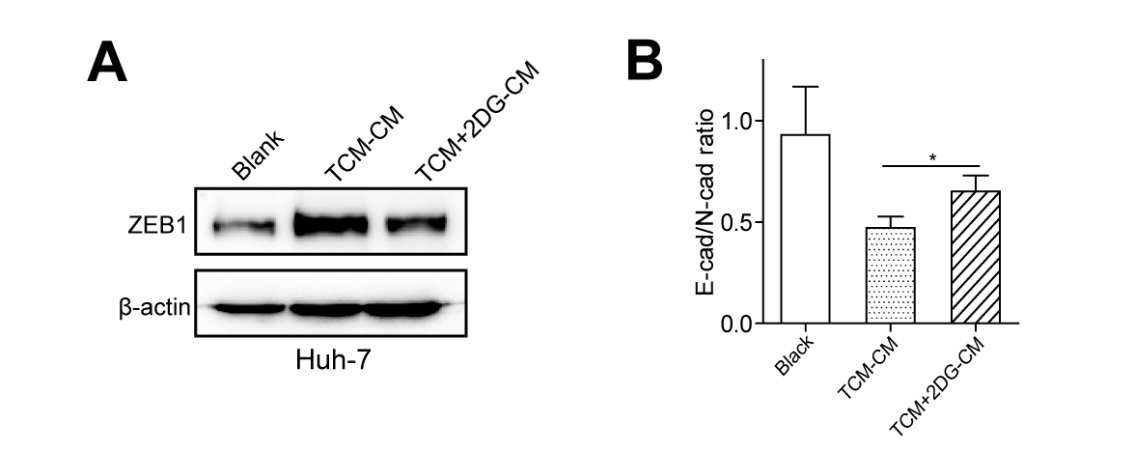


**Supplementary Figure 4. 2DG inhibits TCM-induced EMT-promoting effects of TAMs.** (A) THP-1-M were treated with Huh-7-TCM for 20 h in the presence or absence of 2DG (12.5 mM) to obtain different TAMs. These TAMs were incubated with RPMI 1640 for an additional 24 h to obtain the condition medium (CM). Huh-7 cells were cultured in the presence of indicated CM for 48 h. The expression levels of ZEB1 were determined by western blotting. (B) Statistical analysis of the E-cadhein/N-cadherin ratio in Huh-7 cells from Figure 4F was shown. One representative of at least three independent experiments is shown. 2DG, 2-deoxy-D-glucose; TAMs, tumour-associated macrophages. Data are presented as mean ± SEM from at least three independent experiments (*p < 0.05).


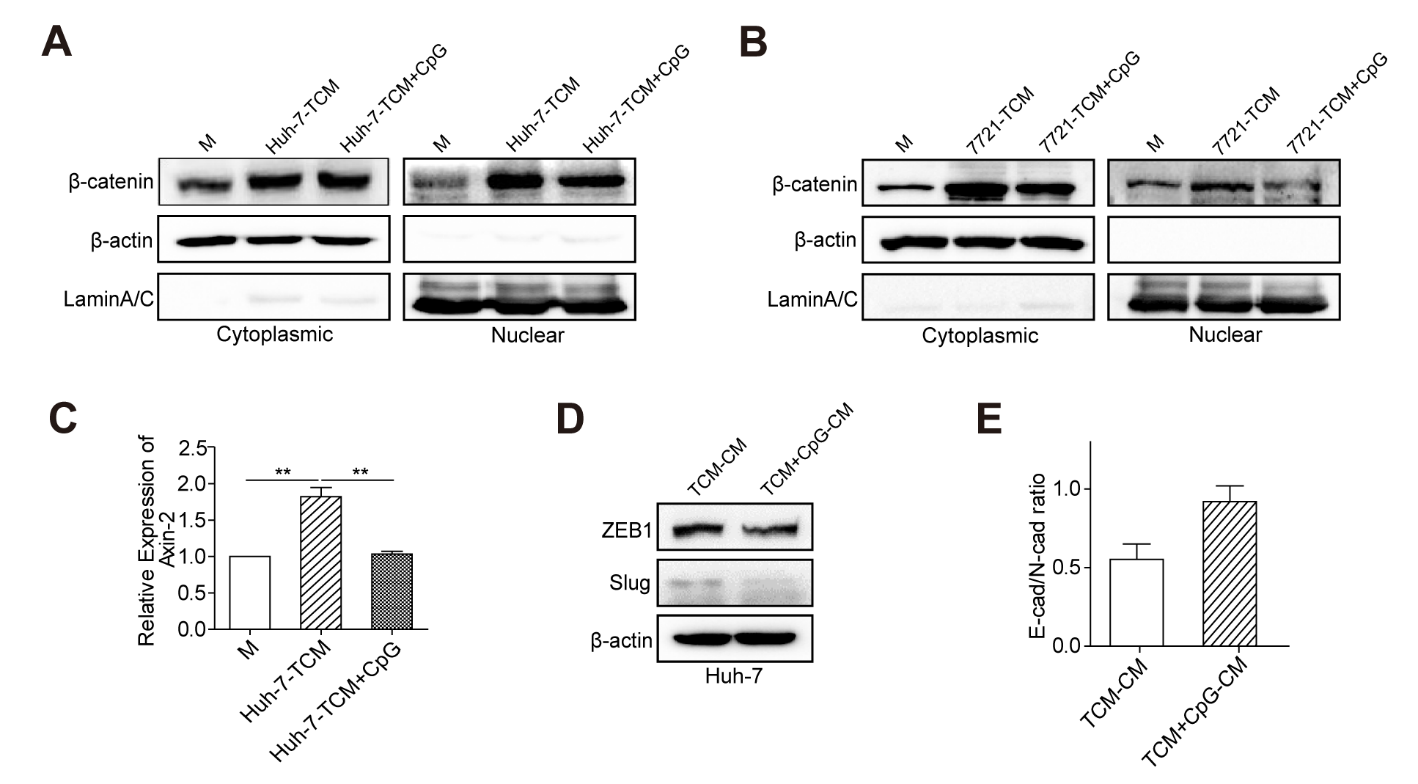


**Supplementary Figure 5. CpG ODN inhibits the activation of Wnt2b/β-catenin signalling in HCC-TAMs and block its EMT promoting effect.** (A, B) THP-1 derived macrophages (THP-1-M) were treated with HCC-TCM for 48 h in the presence or absence of CpG ODN (2 µg/mL). The cytosolic and nuclear proteins in these HCC-TAMs were extracted and β-atenin levels were determined by western blotting. (C) The expression levels of Axin-2 in these TAMs were determined by qPCR. (D) These TAMs were incubated with RPMI 1640 for another 24 h to obtain the condition medium (CM). Huh-7 cells were cultured in the presence of indicated CM for 48 h. The expression levels of ZEB1 was determined by western blotting. (E) Statistical analysis of the E-cadhein/N-cadherin ratio in Huh-7 cells from Figure 5F was shown. One representative of at least three independent experiments is shown. qPCR, quantitative real-time PCR; HCC, hepatocellular carcinoma; TCM, tumour condition culture medium; TAMs, tumour-associated macrophages; 7721, SMMC-7721. Data are presented as means ± SEM from at least three independent experiments (**p < 0.01).


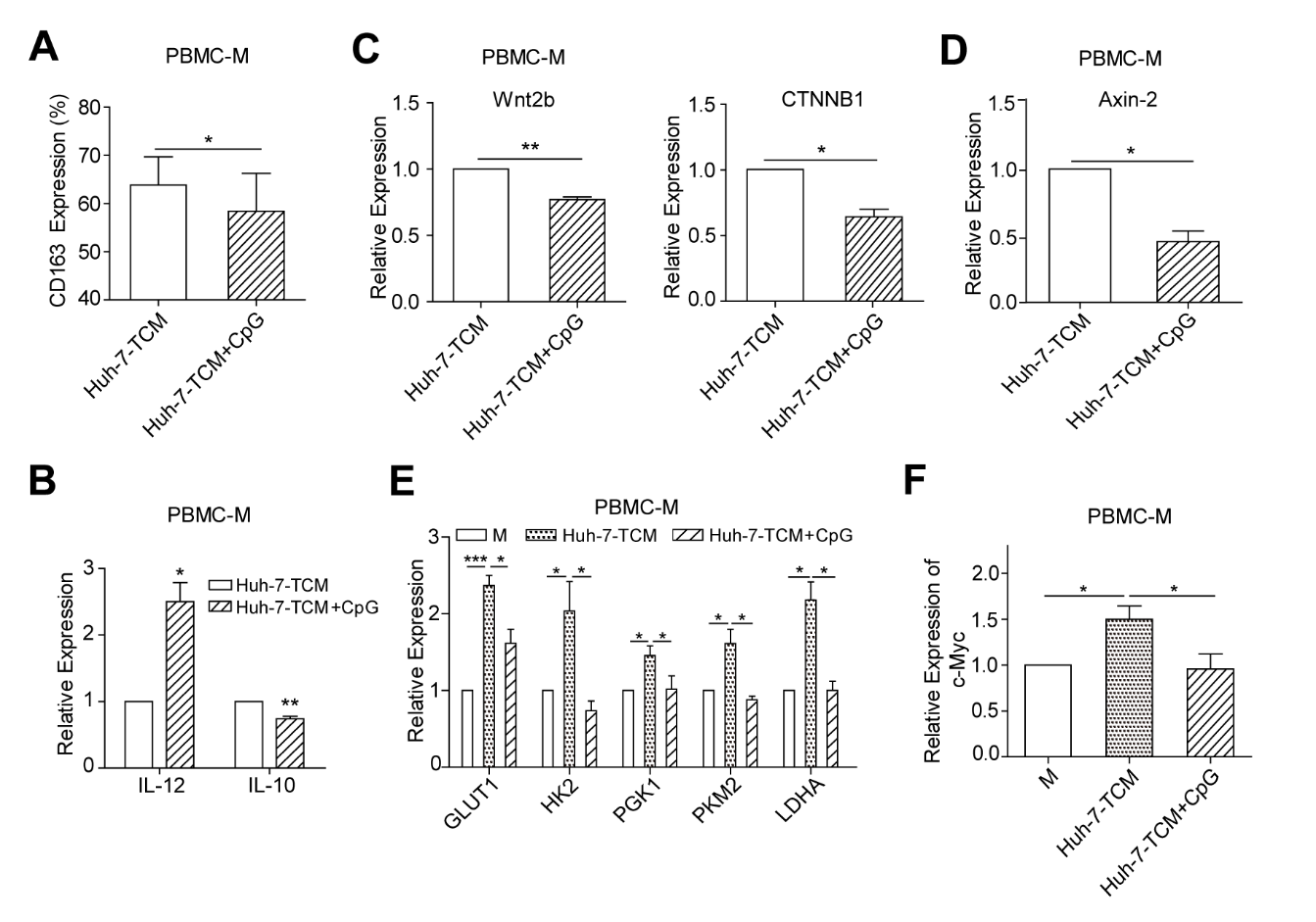


**Supplementary Figure 6. CpG-ODN blocks M2 polarization, downregulates the expression of Wnt2b/β-catenin and suppresses the glycolysis of HCC-TAMs derived from human PBMC-M via c-Myc.** CD14^+^ cells were purified from the peripheral blood of healthy donors and induced with 100 ng/mL M-CSF for 5 days to obtain the PBMC-derived macrophages (PBMC-M). PBMC-M were incubated with 50% Huh-7-TCM for 48 h in the presence or absence of CpG ODN (2 µg/mL). (A, B) The expression levels of CD163 and IL-12, IL-10 on/in these TAMs were determined by flow cytometry and qPCR, respectively. (C,D) The expression levels of Wnt2b, CTNNB1 and Axin-2 in these cells were determined by qPCR. (E) The mRNA expression levels of key enzymes involved in glycolysis were determined in PBMC-M by qPCR. (F) The mRNA expression levels of c-Myc were determined in indicated the PBMC-M by qPCR. One representative of at least three independent experiments is shown. qPCR, quantitative real-time PCR; PBMC, peripheral blood mononuclear cell; HCC, hepatocellular carcinoma; TCM, tumour condition culture medium. Data are presented as mean ± SEM from at least three independent experiments (*p < 0.05, **p < 0.01 and ***p < 0.001).





**Supplementary Figure 7. Factors in HCC-microenvironment that can upregulate Wnt2b expression in tumour-associated macrophages.** THP-1 derived macrophages (THP-1-M) were treated with or without IL-10 (50 ng/mL) or TGF-β (10 ng/mL) for 48 h. The expression levels of Wnt2b, CTNNB1 and c-Myc in these macrophages were determined by qPCR. qPCR, quantitative real-time PCR; HCC, hepatocellular carcinoma; TCM, tumour condition culture medium. Data are presented as mean ± SEM from at least three independent experiments (*p < 0.05).
